# The Attitudes of Patients Toward Orthopaedic Post-surgical Scars

**DOI:** 10.7759/cureus.47975

**Published:** 2023-10-30

**Authors:** Martin P Ho, Hannah Hughes, Patrick Fleming

**Affiliations:** 1 Department of Trauma and Orthopaedic Surgery, Cork University Hospital, Cork, IRL

**Keywords:** posas, scar acceptance, scar appearance, orthopaedics, psycho-social, patient satisfaction, surgical scar

## Abstract

Background

Post-surgical scars (PSS) are an expected consequence of surgery. Several factors have previously been associated with PSS satisfaction including patient age and time elapsed post-operative. Little data are available regarding patient attitudes toward orthopaedic PSS. Knowledge of patient attitudes and the various associated factors may allow physicians to administer peri-operative care to mitigate the potential negative effects of PSS. Our study aims to investigate the attitudes of patients toward their PSS using quantitative scar assessment scales and to identify factors associated with PSS satisfaction.

Methods

We conducted a retrospective study with a follow-up. We included all patients with orthopaedic PSS on their upper or lower limbs between two and 18 weeks postoperative attending Cork University Hospital, Ireland, between February and August 2022. Patients completed an initial baseline questionnaire and then a follow-up questionnaire six months post-operative. The Patient and Observer Scar Assessment Scale (POSAS) evaluated PSS satisfaction. The European Quality of Life 5 Domain (EQ-5D), alongside several Likert scales, evaluated the patient's quality of life (QoL).

Results

In total, 91 patients were included. The mean POSAS score was 28.41 (95% CI, 25.85-30.97). Younger patient age (p=0.045) and decreased time passed post-operatively (p=0.002) were associated with poorer PSS satisfaction. Patients reporting their PSS appearing worse than expected were more likely to agree that their QoL had been adversely affected by it (p=0.001).

Conclusion

Most patients were satisfied with their orthopaedic PSS. This study identified several factors associated with poor PSS satisfaction. Our finding, which associated patient scar expectations and QoL, is novel and has not been previously examined. Accordingly, peri-operative interventions, including scar expectation management, may be implemented to mitigate scar-related QoL impact.

## Introduction

Post-surgical scars (PSS) are an inevitable consequence of surgery. Previous studies have indicated that patients are broadly satisfied with their PSS [1-3] although some may experience a poorer quality of life (QoL) [4].

The Patient and Observer Scar Assessment Scale (POSAS) [5] is a validated, quantitative scoring tool that evaluates aspects of a PSS, including pain and colour. Similarly, questionnaires such as the European Quality of Life 5 Dimension (EQ-5D) [6], as well as Likert scales [7], can quantitatively evaluate a patient’s QoL.

Numerous factors are believed to influence patients’ attitudes towards their PSS, including time elapsed post-operation, patient age, race, and scar location [8,9].

Understanding the factors influencing patient PSS satisfaction facilitates targeted peri-operative interventions that may improve PSS cosmesis, patient satisfaction, and QoL. These include peri-operative discussions managing patient PSS expectations [10] and post-operative treatments [11]. Unfortunately, there is little data available regarding patient attitudes towards orthopaedic PSS.

Our study aims to investigate the attitudes of patients towards their PSS using quantitative scar assessment scales and to identify factors associated with PSS satisfaction.

This article was previously presented as a meeting abstract at the Irish Orthopaedic Association Annual Meeting on June 18, 2022, the 9th University Hospital Waterford Research Meeting on January 20, 2023, and the 48th Sir Peter Freyer Surgical Symposium on September 2, 2023. This article was previously presented as a poster at the Irish Surgical Training Group Research Symposium on November 19, 2022, the 12th International Conference for Healthcare and Medical Students on February 10, 2023, the 2023 Student Medical Summit on February 11, 2023, and the 24th EFORT Annual Congress on May 26, 2023.

## Materials and methods

Inclusion and exclusion criteria

We conducted a retrospective study with a follow-up. We included all patients with orthopaedic PSS on their upper or lower limbs who attended Cork University Hospital (CUH), Ireland, between February and August 2022 and were between two and 18 weeks post-operative.

We excluded patients with diagnosed dementia, patients who underwent orthopaedic surgery at another facility, and patients with PSS in other body locations.

Data collection

Ethical approval was obtained from the Clinical Research Ethics Committee of the Cork Teaching Hospitals (Approval number: ECM 6 (h)). Following approval, the author attended CUH and accessed patient notes to identify patients who met the inclusion criteria and were due to attend that day.

**Figure 1:**
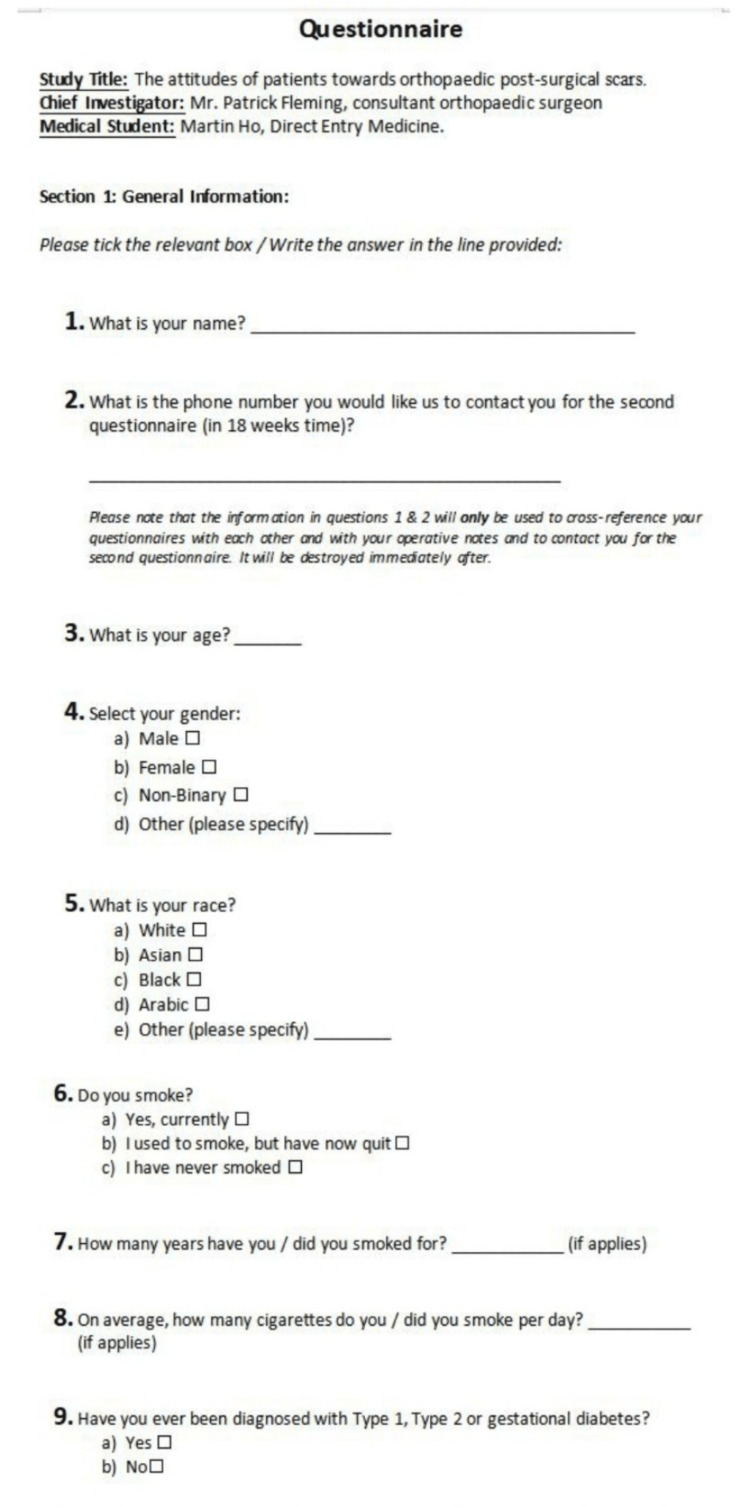
The General Information Questionnaire

**Figure 2:**
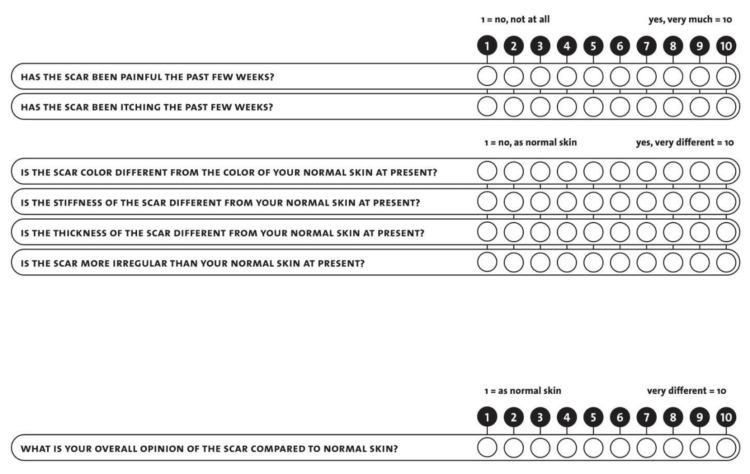
The Patient Section of the Patient and Observer Scar Assessment Scale (POSAS) To assess PSS satisfaction, the author obtained permission to use the Patient and Observer Scar Assessment Scale (POSAS) [5]. Link to the original author's website: https://www.posas.nl/

**Figure 3:**
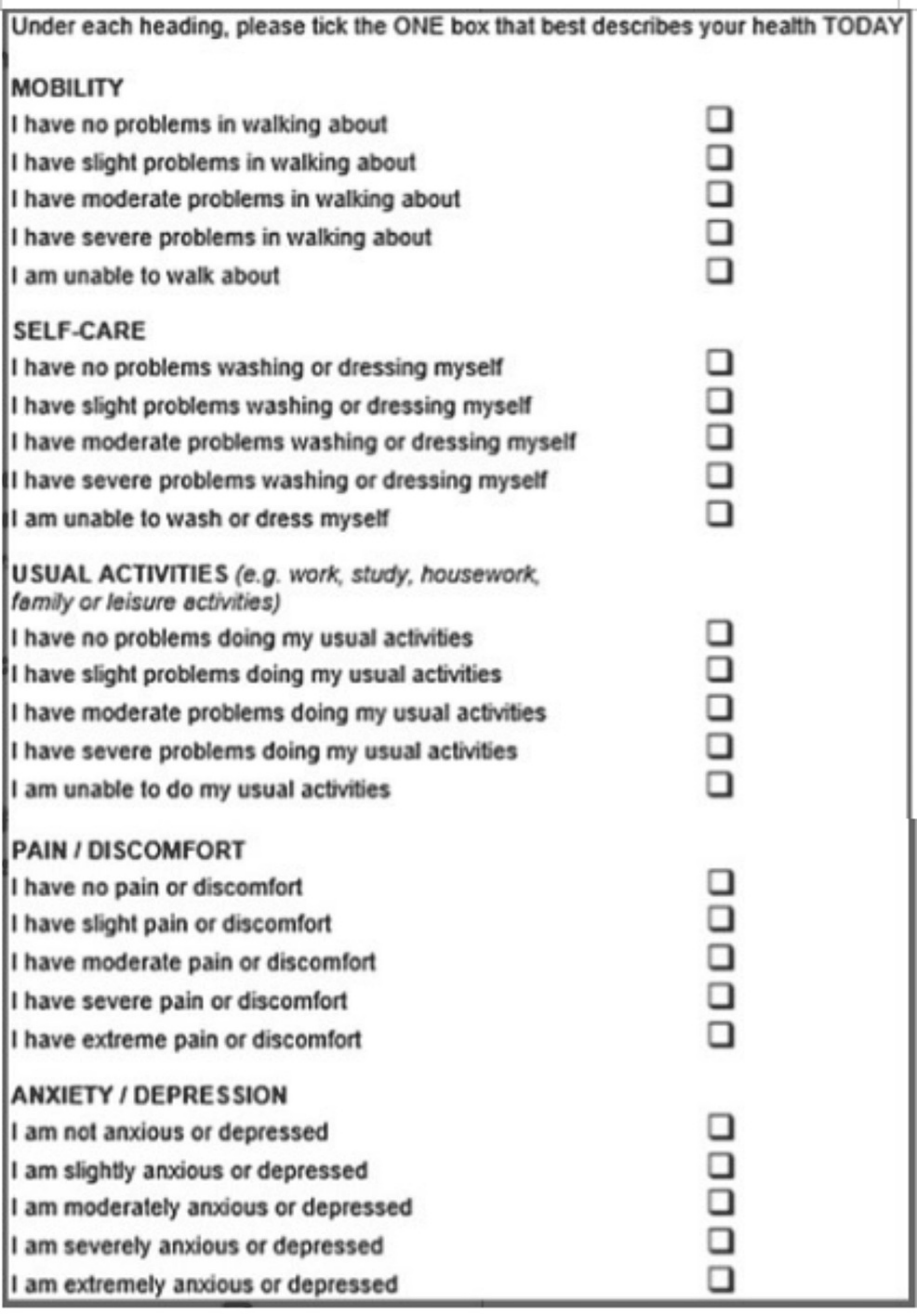
The European Quality of Life 5-Domain (EQ-5D) Questionnaire To assess the QoL impact of PSS, the author obtained permission to use the EQ-5D Questionnaire [6]. Link to the original website: https://euroqol.org/eq-5d-instruments/eq-5d-5l-about/

**Figure 4:**
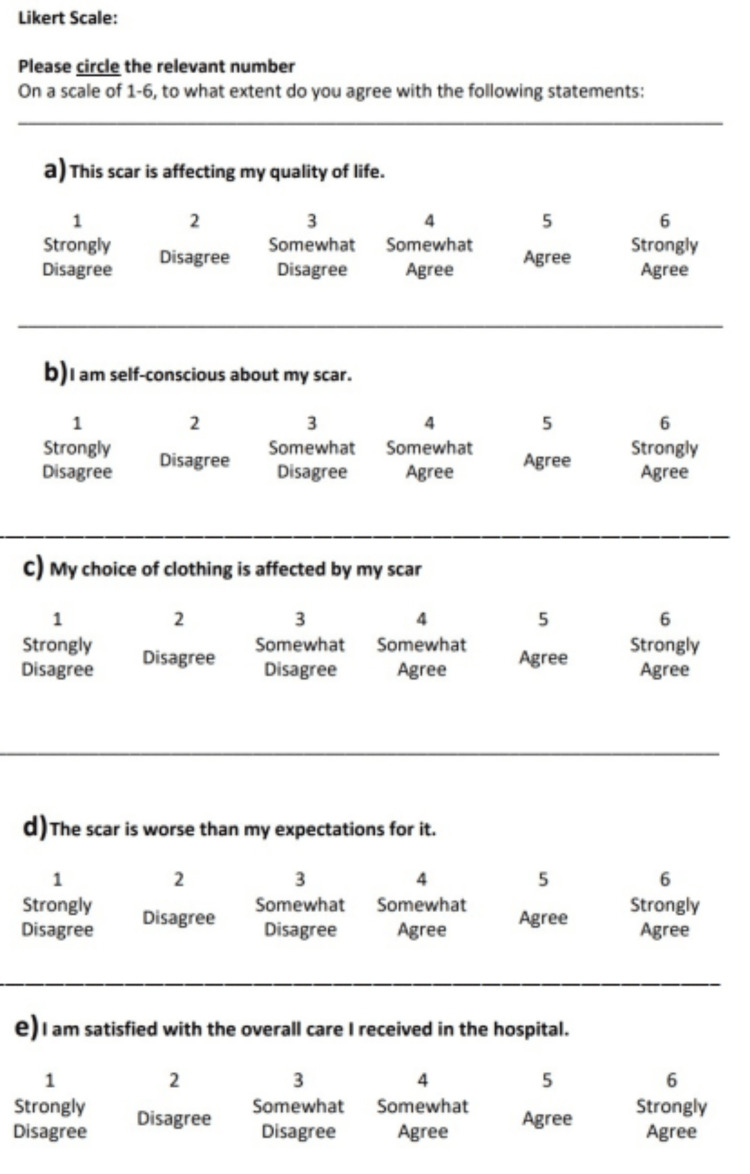
Likert Scale Questionnaire

The patient completed the following questionnaires: A general information questionnaire (which included consent to participate) (Figure 1), the patient section of the Patient and Observer Scar Assessment Scale (POSAS) (Figure 2), the European Quality of Life 5 Dimension Scale (EQ-5D) (Figure 3), and several Likert scales (Figure 4).

The questionnaires were administered to patients on two occasions: between two and 18 weeks post-operative (in-person at the CUH orthopaedic fracture clinic, baseline questionnaire) and at six months post-operative (via telephone).

Retrospective chart review

After completion of the initial questionnaire, the following data were collected from participant post-operative notes: Date of operation, operation type, closure method (presence/absence of clips), presence/absence of surgical complications, and scar location.

Questionnaire design

To assess PSS satisfaction, the author obtained permission to use the POSAS questionnaire. As this study only examined patient PSS satisfaction, the “Observer” section was omitted. The total score ranged from six to 60 with a lower score indicating a better scar.

To assess the QoL impact of PSS, the author obtained permission to use the EQ-5D questionnaire. The EQ-5D score ranged from -0.594 to +1 with a higher score indicating a better QoL.

To further assess QoL impact, several six-part Likert scales were designed by the authors. They asked questions pertinent to the objectives of the study that were not addressed in other scar assessment scales. Similar Likert scales have been used in previous surgical literature [12].

Data analysis

All statistical analysis was performed using Statistical Package for the Social Sciences (SPSS) version 28 (IBM Corp., Armonk, New York). Descriptive analysis was performed on patient demographic characteristics, smoking and diabetic status, operation-related variables, and Likert scale responses. Values pertaining to PSS satisfaction (POSAS and EQ-5D results) were reported as the mean and 95% confidence intervals. Regression analysis investigated the association between several factors and patient POSAS, EQ-5D, and Likert scale responses. Spearman's rank correlation coefficient evaluated the internal correlation of Likert scale responses.

## Results

Descriptive analysis

N=91 patients were included. Fifty-three (58.24%) were female and 38 (41.76%) were male. The mean patient age was 47.87+/-19.8 years (Range 14-79). The mean scar age at the initial questionnaire was 8.98 weeks (+/-5.3 weeks). 86 (94.5%) patients were white, three were black, and two were Asian. Forty-two patients (46.15%) reported being current or ex-smokers while 49 (53.85%) stated they never smoked. Six patients (6.59%) had been diagnosed with either type 1, 2, or gestational diabetes while 85 (93.41%) had not (Table 1).

**Table 1:** Patient demographic characteristics and health status

Population	n	Standard Deviation	%
Gender			
Male	38		41.76
Female	53		58.24
Total	91		-
Age			
Mean patient age	47.87	+/- 19.8	-
Mean scar age (weeks)	8.98	+/- 5.3	
Race			
White	86		94.5
Black	3		3.30
Asian	2		2.20
Smoking status			
Current / ex-smoker	42		46.15
Never smoked	49		53.85
# Pack year			
Mean (years)	16.76	+/-17.92	
Diabetic status			
Type 1/2/GDM	6		6.59
No Diabetes	85		93.41

Surgical characteristics

The most common operation performed was Open Reduction Internal Fixation (ORIF) (n=65, 72.22%), followed by K-Wire (n=9, 9.1%) and Intramedullary Nail (IM Nail) Insertion (n=6, 6.59%). The most common operative site was the leg (n=46, 50.55%), followed by the forearm (n=22, 24.18%), shoulder (n=15, 16.48%), and hip (n=8, 8.79%). According to the post-operative notes, seven patients (7.7%) had surgical complications. 26 patients (28.57%) had their incisions closed with clips (Table 2).

**Table 2:** Surgical characteristics of patients ORIF: open reduction internal fixation; IM: intramedullary nail

	n	%
Operation		
ORIF	65	72.22
IM Nail	6	6.59
K-Wire	9	9.10
Other / Mix	11	12.09
Location		
Forearm	22	24.18
Shoulder	15	16.48
Leg	46	50.55
Hip	8	8.79
Surgical Complications		
Yes	7	7.69
No	84	92.31
Closure With Clips		
Yes	26	28.57
No	65	71.43

Follow up

N=61 patients completed the second questionnaire, with 30 patients (33%) being lost to follow-up. There were no significant differences in patient demographics or mean POSAS (p=0.771) and EQ-5D (p=0.131) scores in the participants lost to follow-up.

Data analysis

**Figure 5:**
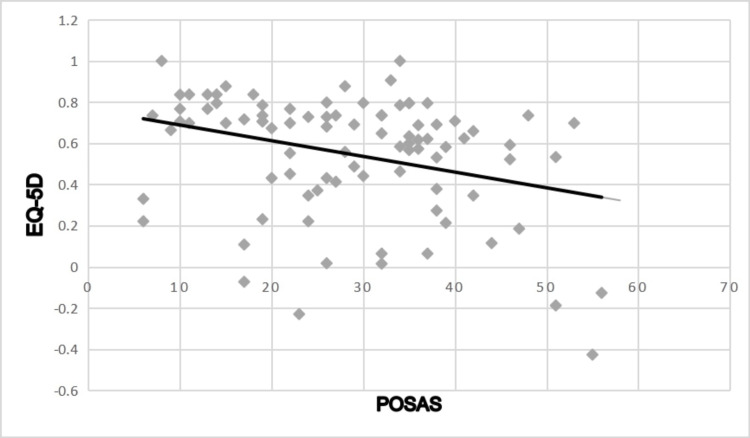
Correlation between POSAS and EQ-5D scores r=-.317, p=0.002 EQ-5D: European Quality of Life 5 Domain; POSAS: Patient and Observer Scar Assessment Scale

The mean POSAS score at the initial outpatient visit was 28.41 (95% CI, 25.85-30.97). This improved to 18.67 (95% CI, 16.8-20.5) at six months post-operative (p<0.0001). The mean EQ-5D score also improved from 0.547 (95% CI, 0.49-0.61) to 0.747 (95% CI, 0.69-0.81) at six months post-operative (p<0.0001) (Table 3). Pearson’s correlation coefficient revealed a significant correlation between patient POSAS and EQ-5D scores (r=-0.317, p=0.002) (Figure 5).

**Table 3:** Differences in POSAS and EQ-5D scores between the initial baseline questionnaire and six months post-operative EQ-5D: European Quality of Life 5 Domain; POSAS: Patient and Observer Scar Assessment Scale

	Initial questionnaire (2-18 weeks) n=91		6 months n=61		
	Value 95% CI		Value 95% CI		p
Mean POSAS (6 to 60)	28.41	25.85-30.97	18.67	16.8 to 20.5	<0.0001
Overall Opinion (1 to 10)	5.64	5.09 to 6.18	4.92	4.31 to 5.53	0.0918
EQ5D (-0.594 to 1)	0.547	0.487 to 0.607	0.747	0.687 to 0.807	<0.0001

Likert scale analysis

**Figure 6:**
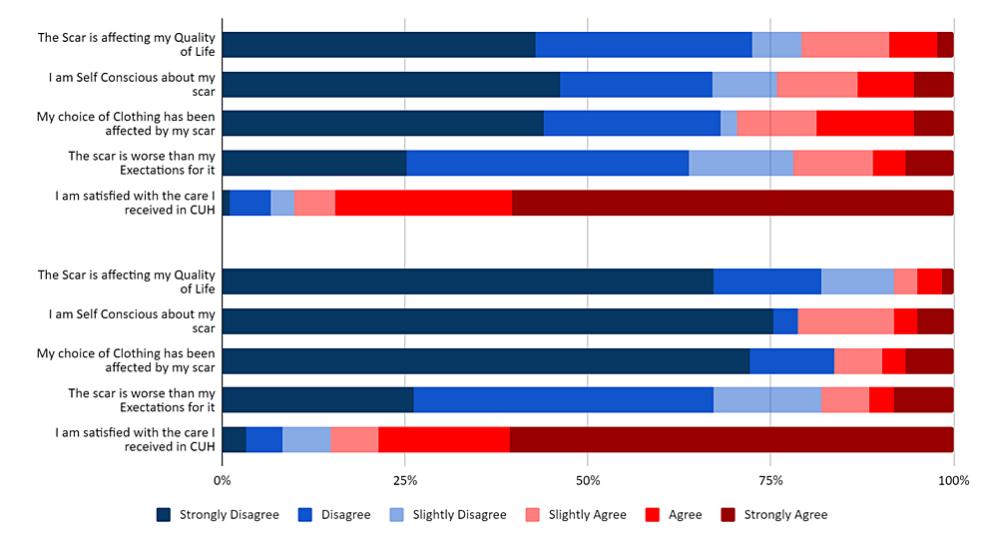
Likert scale results

In the initial questionnaire, 20.88% of patients (n=19) agreed with the statement, “The scar is affecting my quality of life.” At six months post-operative, only 8.2% of patients (n=5) agreed with the statement. Initially, 21.98% of patients (n=20) agreed with the statement, “The scar is worse than my expectations for it”. In the second questionnaire, 18.03% of patients (n=11) agreed with the statement (Figure 6).

Patients who believed that the scar was worse than their expectations were more likely to feel self-conscious about their scar (Rho=.433, 95% CI, .250-.587, p<0.001), more likely to have their choice of clothing affected by it (Rho=.410, 95% CI, .233-.568, p<0.001), and more likely to report a poorer quality of life (Rho=.568, 95% CI, .410-.693, p<0.001). They were also more likely to rate their scar poorly on the POSAS questionnaire (Rho=.446, 95% CI, .263-.598, p<0.001).

Factor analysis

Patient Age

**Figure 7:**
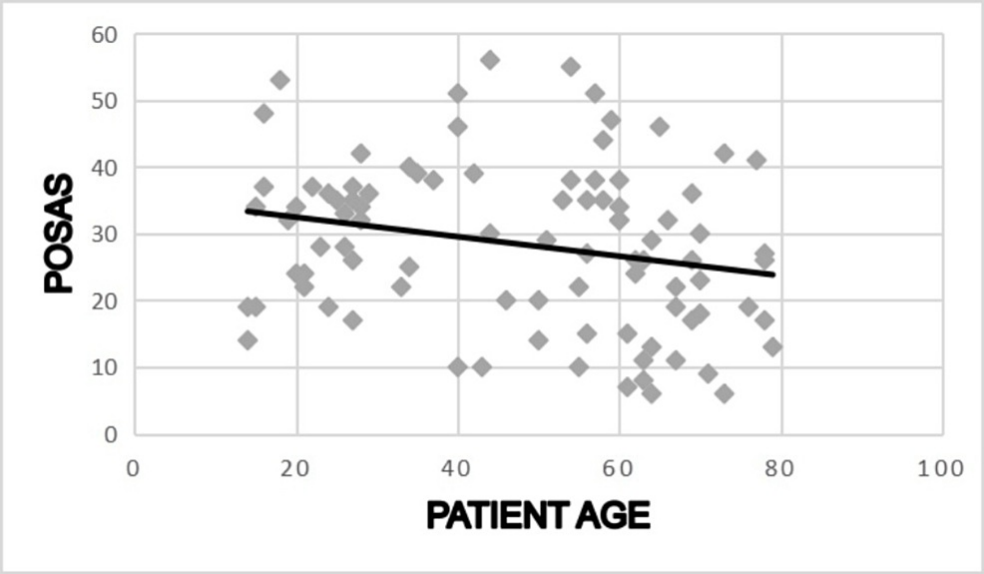
Correlation between patient age and POSAS scores for n=91 patients Rho=-.237, p=0.024 POSAS: Patient and Observer Scar Assessment Scale

In the initial questionnaire, younger patient age was associated with poorer POSAS scores compared to older patient age (Rho=-.237, 95% CI, -.031 - -.423, p=0.024) (Figure 7). However, at six months post-operative, this association was no longer seen (r= -0.052, p=0.692).

Scar Age

**Figure 8:**
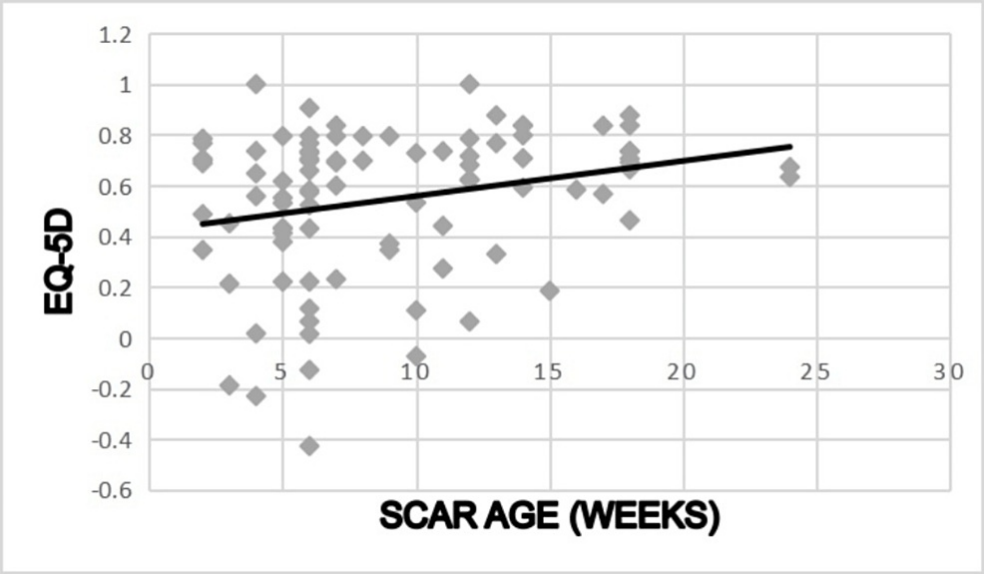
Correlation between scar age and POSAS scores Rho=.249, p=0.018 POSAS: Patient and Observer Scar Assessment Scale

Younger scar age was associated with poorer EQ-5D scores (Rho=.249, 95% CI, .044-.434, p=0.018) (Figure 8).

Surgical Complications

**Figure 9:**
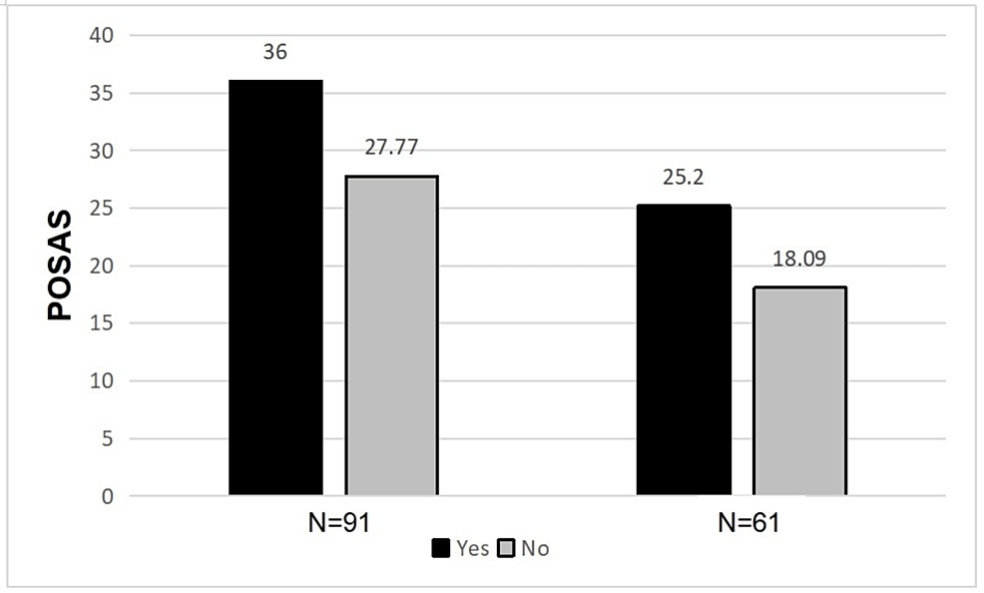
Difference in mean POSAS scores between patients with and without surgical complications POSAS: Patient and Observer Scar Assessment Scale

**Figure 10:**
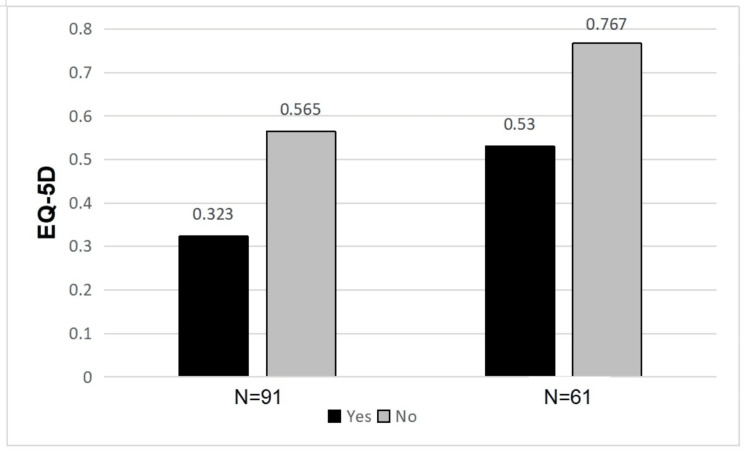
Difference in mean EQ-5D scores between patients with and without surgical complications EQ-5D: European Quality of Life 5 Domain

There was a statistically significant difference in the mean POSAS and EQ-5D scores between patients with surgical complications (p=0.043, p=0.019) and those without (Figures 9, 10). This difference also existed at six months post-operative (p=<0.001, p=<0.001).

No association was found between the following variables and POSAS or EQ-5D scores: Patient gender (p=0.186, p=0.447), patient race (p=0.489, p=0.641), patient diabetic status (p=0.131, p=0.07), closure method (with/without clips) (p=0.16, p=0.203), and operation type (p=0.641, p=0.883).

Scar Location

**Figure 11:**
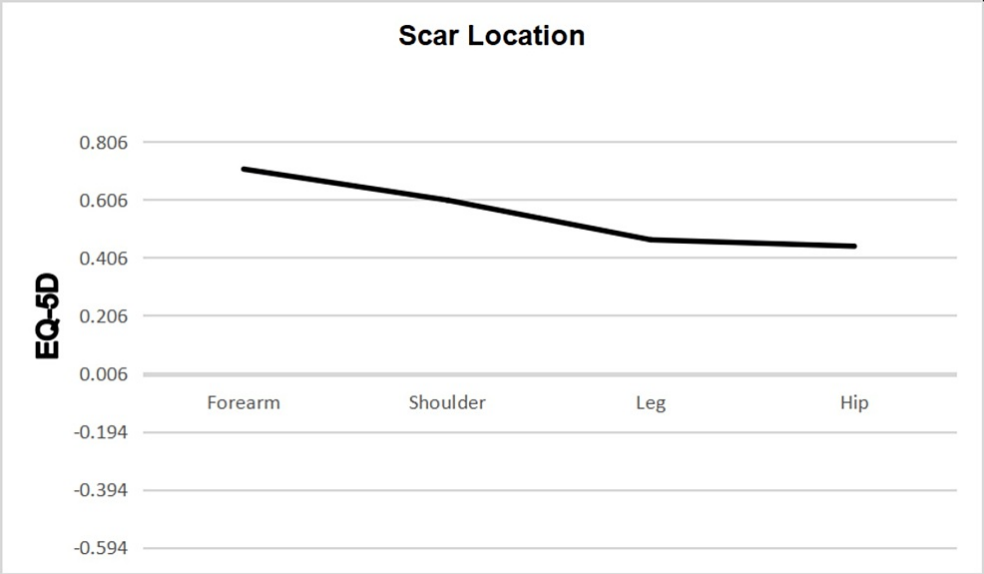
Differences in mean EQ-5D scores between scar locations EQ-5D: European Quality of Life 5 Domain

No association was found between scar location and POSAS scores (p=0.897). An association was found between scar location and EQ-5D scores (p=0.006) (Figure 11).

## Discussion

Most patients were satisfied with their PSS, according to the POSAS scores. This satisfaction increased between the initial baseline questionnaire and the follow-up telephone questionnaire at six months post-operative. These findings allow physicians to educate and reassure patients about the expected healing benefits of time.

Similarly, most patients reported a favourable QoL, according to the EQ-5D and Likert scales. An improvement in QoL was also observed between the initial visit and six months post-operative. This knowledge can be equally motivating for patients. Moreover, it can guide physicians in determining the appropriate timing for interventions that mitigate QoL impact. Interventions such as scar massage therapy and moisturisation may be recommended shortly after the operation, whereas scar revision surgery may be considered at a later stage when QoL improvement is not anticipated physiologically [11].

Pearson’s correlation coefficient revealed a significant correlation between patient POSAS and EQ-5D scores (Rho=-.317, p=0.002) indicating patients who rated their scars poorly were more likely to report a poorer QoL. This underscores the need for physicians to prioritise PSS satisfaction.

In the initial questionnaire, younger patient age (p=0.024) was associated with poorer POSAS scores. This is consistent with established surgical literature from other specialities [8,13]. Therefore, it is appropriate to consider a lower threshold for interventions in this population to achieve improved cosmetic outcomes.

We did not find an association between patient gender and mean POSAS or EQ-5D scores. This is consistent with previous scar satisfaction literature [8,14-16] and contradicts widely held gender stereotypes. It is important that physicians are aware of this to avoid male patients with scar concerns being overlooked.

Unmet patient expectations for their PSS were associated with poorer POSAS scores (p<0.001), as well as poorer QoL according to the Likert scale (p<0.001). This association has not been previously examined within PSS satisfaction literature. It highlights the potential significance of patient scar expectations as a determinant of post-operative QoL outcomes, therefore warranting greater attention and examination.

Limitations

In total, 30 patients (32.97% of the original sample) did not complete the second questionnaire. This may be attributed to participants’ reluctance to answer calls from unknown numbers.

While the EQ-5D is a validated tool to measure QoL in patients, it did not specifically assess the impact of PSS on patient QoL. Accordingly, its results may have been confounded by the effects of the initial patient injury on patient QoL. However, we included several Likert scales that specifically addressed the impact of PSS on patient QoL.

Future recommendations

Research Recommendations

This study represents the first examination of patient attitudes toward orthopaedic PSS. To strengthen its findings, future studies should be conducted in an ethnically diverse population.

Furthermore, given the personal nature of scar satisfaction, qualitative studies may provide a deeper understanding of patient perceptions.

Clinical Recommendations

Following our novel association between patient PSS expectations and QoL, we recommend that scar expectation discussions become part of the pre-operative consultation and consent process. This ensures that patients are not surprised by their scar cosmesis post-operatively. This may improve patient PSS satisfaction and QoL [17]. The length and depth of such discussions should be guided by the patient’s initial thoughts and concerns about a future PSS, as well as the patient’s age.

Furthermore, we recommend that efforts be made to minimise surgical complications, such as wound infection, as they have been associated with poorer PSS satisfaction. Strategies to decrease complications may include the utilisation of surgical safety checklists and emphasis on proper tissue handling peri-operatively [18].

## Conclusions

Most patients are satisfied with their orthopaedic PSS. This study identified several factors associated with poorer PSS satisfaction, including younger patient age, younger scar age, and the presence of surgical complications. This is consistent with established surgical literature. A novel finding of this paper is the association between pre-operative scar expectations and post-operative QoL. Accordingly, peri-operative interventions can be implemented to mitigate the negative QoL effects of PSS.
